# A High-Performance Ordered Routing Algorithm for Large-Scale WLCSP with Multi-Capacity †

**DOI:** 10.3390/mi16121352

**Published:** 2025-11-28

**Authors:** Chuandong Chen, Dishi Lin, Qinghai Liu, Zhifeng Lin

**Affiliations:** 1 School of Microelectronics, Fuzhou University, Fuzhou 350108, China; cdchen@fzu.edu.cn; 2 College of Computer and Data Science, Fuzhou University, Fuzhou 350108, China; lindisi2000@outlook.com; 3 Center for Discrete Mathematics and Theoretical Computer Science, Fuzhou University, Fuzhou 350108, China; qliu@fzu.edu.cn; 4 School of Mathematics and Statistics, Fuzhou University, Fuzhou 350108, China

**Keywords:** fan-out WLCSP, redistribution layer, multi-capacity MMCF, pre-assignment routing

## Abstract

Redistribution layer ordered routing is a critical problem in fan-out wafer-level chip-scale packaging (WLCSP) design. The traditional integer linear programming (ILP) method is inefficient in dealing with the ordered routing problem of multiple-capacity. Hence, we propose a high-performance ordered routing algorithm to solve the multiple-capacity ordered routing problem on the redistribution layer (RDL). First, we transform the ordered routing problem into the min-cost multi-commodity flow (MMCF) problem and use the linear programming (LP) method to solve it. Then, we use depth-first search (DFS) to process the LP method flow results and obtain the pre-assignment I/O candidate paths. Finally, the candidate path set obtains legal routing results by setting the crossing weight and a heuristic algorithm to receive the minimum crossing weight. When the pre-assignment I/O routing is uncompleted, we will set the capacity of tile nodes and edges to 0 and perform iterative routing for better results. Compared with the state-of-the-art work, experimental results show that our algorithm can solve twice the scale of the RDL ordered routing problems and reduce the routing time by 17% when dealing with multi-capacity RDL ordered routing problems.

## 1. Introduction

**Figure 1:**
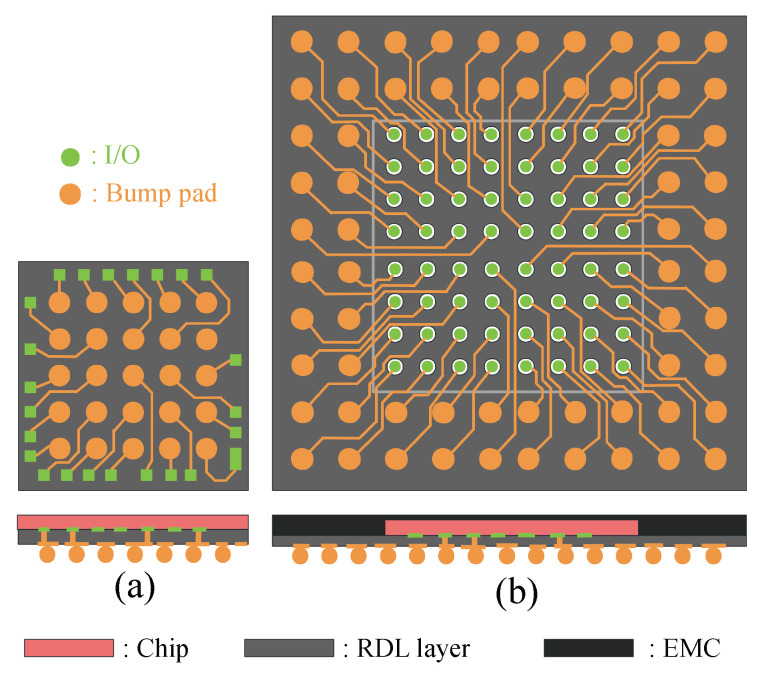
A 5 × 5, 0.4 mm pitch fan-in WLCSP from a wire bond device’s perimeter bond pads (**a**) and a one-layer fan-out WLCSP design from an 8 × 8, 0.3 mm pitch device to a 0.4 mm pitch package (**b**).

For modern integrated circuit (IC) design, the increase in complexity and the reduction in feature size have led to the need for higher-density distribution of I/Os [1,2], which has become a significant issue facing packaging technology. WLCSP can achieve high-density integration and miniaturization of chips [3,4,5]. WLCSP is an advanced technology that offers several advantages, including a small size, excellent electrical performance, effective heat dissipation, and low cost. In WLCSP, the I/Os can be distributed along the four sides of the chip or arranged in an array, as shown in Figure 1. WLCSP are divided into fan-in (Figure 1a) and fan-out (Figure 1b) packages based on bump pad distribution. When routing I/O in WLCSP, internal I/Os are routed to bump pads through the RDL. The routing area is divided into two regions: the fan-in area, which is the area inside the chip, and the fan-out area, which is the routable area beyond the chip’s physical size [6].

According to different routing requirements, the fan-out WLCSP ordered routing is divided into (1) free-assignment (FA) routing; (2) pre-assignment (PA) routing; (3) unified-assignment (UA) routing [7]. For FA routing, the connection relationship between I/Os and bump pads is not defined before routing. The I/Os are assigned to any bump pad in the routing process. For PA routing, the routing order of I/Os is pre-defined before routing. For UA routing, some assignments from I/Os to bump pads are pre-defined, while others are not. The routing results of PA and UA routes must follow a specific order, whereas FA do not. However, disordered routing paths will make subsequent chip connections difficult. Therefore, both the PA routing for a single chip and the UA routing for multiple chips are superior to the FA. The fan-out WLCSP I/O ordered routing problem discussed in this paper is the PA routing problem of array I/O distribution.

### 1.1. Previous Work

Fang et al. [8] proposed an algorithm based on network flow to solve the FA routing problem. Fang et al. [9] proposed an ILP-based algorithm to complete the PA routing problem. Lin et al. [10] developed an efficient dynamic programming iterative algorithm based on net sequence switching. Chen [11] utilized machine learning to accelerate the solution of the ordered routing problem by removing routing grids with low occupancy probability, thereby narrowing the solution space in fan-out WLCSP. These methods for the ordered routing problem are ILP methods. Limited by NP-hard (Non-deterministic Polynomial-time hard), as the number of pre-assignment I/Os increases, routing capacity increases, and as the scale of the fan-out WLCSP I/O ordered routing flow model expands, the ILP method encounters problems such as uncontrollable routing length, excessive time, and no solution. Therefore, we propose a faster and higher-performance algorithm to solve the large-scale fan-out WLCSP ordered routing.

### 1.2. Our Contributions

We summarize our main contributions as follows:

- We use the MMCF model and LP method to solve the fan-out WLCSP I/O ordered routing problem. The LP method has polynomial time complexity, which is faster than the traditional ILP solver, for dealing with ordered routing problems. Then, we use the DFS to process the LP flow results to obtain candidate paths.
- We propose a crossing weight heuristic algorithm to solve the problem of path crossings. It can quickly obtain legal routing results that conform to the ordered routing constraints. We set the capacity of tile nodes and edges that have completed the routing path to 0. It solves the problem where the follow-up path crosses the completed routing path in iterative routing.
- Experimental results demonstrate that our algorithm can solve fan-out WLCSP ordered routing problems with twice the scale and reduce routing time by 17% when handling multi-capacity ordered routing problems. The performance advantage of our algorithm becomes more evident as the routing capacity and scale increase.

The rest of this paper is organized as follows. Section 2 gives some preliminaries and the problem statement. Section 3 introduces our algorithm in detail. Experimental results are shown in Section 4. Finally, Section 5 concludes our work, and Section 6 presents the outlook. We summarize the notations and abbreviations used in this paper as in Table 1.

## 2. Preliminaries

### 2.1. Problem Formulation

The ordered routing problem of fan-out WLCSP can be stated as follows.

Given: m×n I/O grid array *R* and a set of I/Os with specific orders *P*, I/Os are regularly arranged in a series of square grids, bumps are evenly distributed around the chip for pre-assignment I/O connections, and the routing area consists of (m+2)×(n+2) routing grids.

Find: The goal of the ordered routing problem is to successfully allocate all I/Os in the set of *P* to the boundary bump while satisfying all the constraints. Subject to:

1. Different I/O paths cannot cross each other.
2. When routing, I/O must follow the preset order to reach the boundary bumps.
3. The capacity consumed when routing must be less than the set tile node and tile edge capacity limits.

Objective: Ensure successful routing of as many pre-assignment I/Os as possible and minimum total routing length and running time.

We divide the fan-out WLCSP I/O ordered routing problem into three parts: Model Construction, Preliminary Routing, and Path Choosing. Model Construction mainly affects routing scenarios and schemes. Preliminary Routing mainly affects the number and allocation of candidate routes. Path Choosing mainly affects the final routing result.

### 2.2. Ordering Constraints

**Figure 2:**
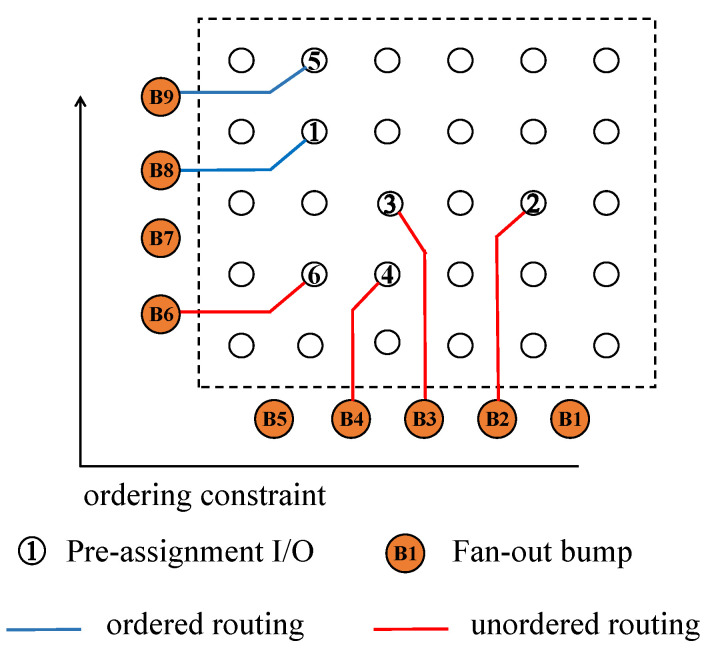
Ordering constraint diagram.

Ordering constraints are the most crucial constraints of the fan-out WLCSP I/O ordered routing problem. Suppose the bump Bi is reached by I/O *i* and the bump Bj is reached by I/O *j*. If for any I/O j<i, Bj<Bi, then the I/O *i* is called ordered routing. Otherwise, if there exists an I/O j<i but Bj>Bi, then the I/O *i* does not satisfy the ordered routing constraint. As shown in Figure 2, we define the ordering constraint as clockwise. I/O 6 violates the ordering constraint because B9>B6. I/O 1 and I/O 5 satisfy the ordering routing constraint. Although there is no problem with I/O 1, we will rip-up and reroute I/O 1 to increase routability.

### 2.3. Min-Cost Multi-Commodity Flow Model (MMCF Model)

**Figure 3:**
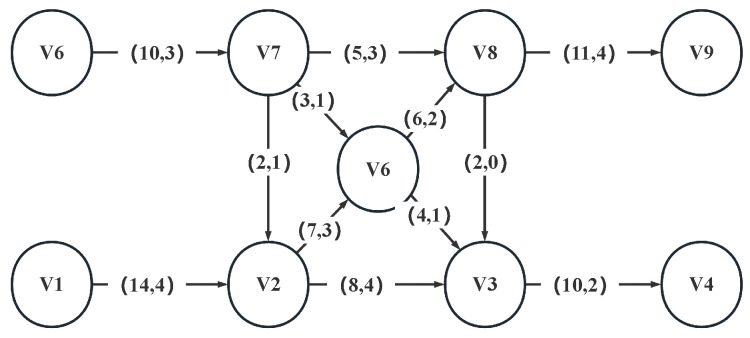
Min-cost multi-commodity flow diagram.

The MMCF is a network flow model [12] in which multiple items, or commodities, flow from various sources to different sinks in the network at minimum cost. The MMCF is shown in Figure 3. Ssources like V1 and V6 flow to sinks like V9 and V4. For Figure 3’s directed edge (14, 4), 14 represents the remaining capacity, and 4 represents the cost of each commodities unit passing through this edge. The goal is to find a set of paths that can transport the most commodities at the lowest possible cost.

## 3. Our Algorithm

**Figure 4:**
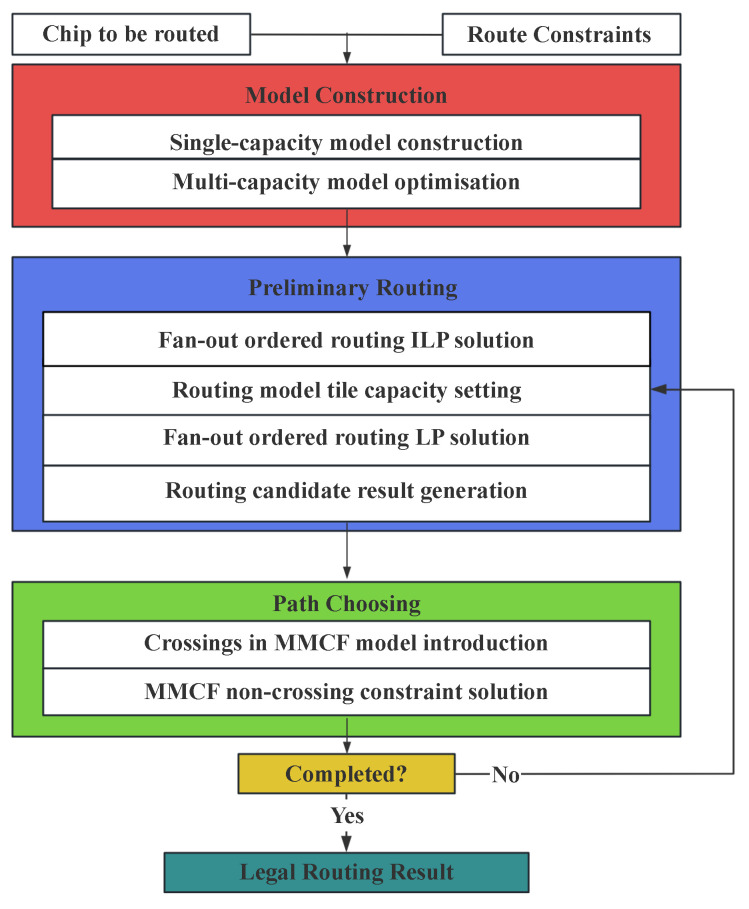
Overview of our algorithm.

Figure 4 summarizes the overall process of our algorithm. First, we transform the ordered routing problem into an MMCF problem. Then, we use the LP method to solve the ordered routing problem and employ DFS to obtain the set of candidate paths for the pre-assignment I/O. Finally, the pre-assignment I/O routing results are selected by a crossing weight heuristic algorithm. After setting the capacity of tile nodes and edges that have completed the routing path to 0, we perform iterative routing until all pre-assignment I/Os are routed and the final routing results meet the ordered routing constraints.

### 3.1. Model Construction

This section mainly discusses the construction of the fan-out WLCSP I/O ordered routing model. We first explain the structure of the single-capacity model and then how to optimize it into a multi-capacity model. Finally, we present the detailed structure of the multi-capacity model, along with its related instructions.

#### 3.1.1. Single-Capacity Model Construction

**Figure 5:**
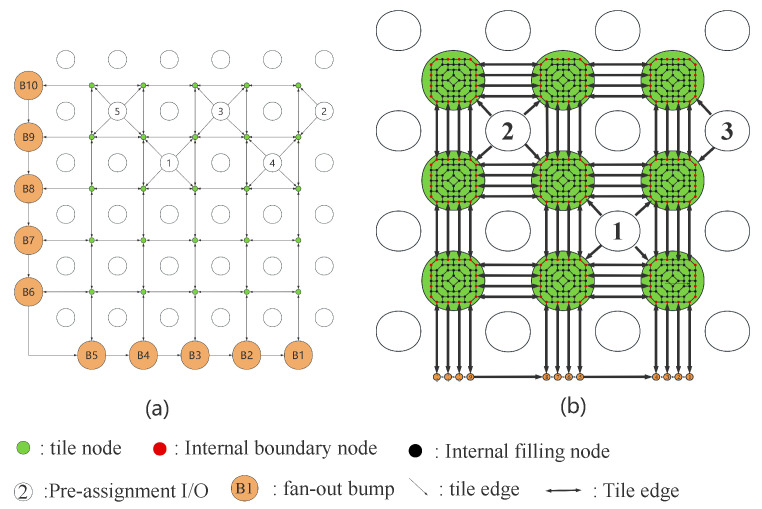
(**a**) Single-capacity fan-out WLCSP I/O ordered routing model. (**b**) Multi-capacity fan-out WLCSP I/O ordered routing model with a capacity of 4.

The single-capacity model in the ordered routing problem is shown in Figure 5a. The m×n I/Os comprise *m* rows and *n* columns and are “arrayed” in square “grids”. We add a tile node (green circle in Figure 5a) to the center of the square grid. The tile edge connects the adjacent tile nodes. We define all I/Os that must be allocated as pre-assignment I/Os (white circle with number in Figure 5a). The pre-assignment I/O as the source point has four directed edges that point to the surrounding tile node. We call the vertex at the boundaries a fan-out bump (orange circle in Figure 5a). All fan-out bumps are numbered clockwise and sequentially connected in descending order. The tile node closest to the fan-out bump has a directed edge that points to the fan-out bump. We assign a cost *c* and capacity $a^{e}$ to each edge and a node capacity $a^{r}$ to all tile nodes. This way, we obtain a directed graph G(D,E,C,V) for ordered routing problems. The directed graph G(D,E,C,V) is a network flow model, and all directed edges are feasible paths for ordered routing.

#### 3.1.2. Multi-Capacity Model Optimization

For the multi-capacity fan-out WLCSP I/O ordered routing model, we optimize the tile node and edge in the network flow model. Figure 5b shows the ordered routing flow model with capacity 4 and the detailed structure of a tile node.

In the detailed structure of the multi-capacity model, we divide tile node *u* into $u_{b}$ and $u_{in}$. The internal boundary nodes of tile node *u* are $u_{b}$ (red circle in Figure 5b). The internal filling nodes $u_{in}$ (black circle in Figure 5b) between the internal nodes of the tile node are the internal filling nodes that are not connected to other tile nodes or fan-out bumps. The internal edges $e_{in}^{u}$ of tile node *u* are connected by internal nodes $u_{b}$ and $u_{in}$.

The capacity of internal nodes and internal edges is the same as that of the tile node and tile edge. We set the cost of the internal edge $e_{in}^{u}$ as $Q\ll c^{e^{qr}}$ based on three points. First, the cost of the internal edge is determined by the distance between the internal nodes. The internal nodes are adjacent to each other, and the distance between them is minimal, so the cost of the internal edge is also a minimum value. Second, the influence of internal edges is far less than that of tile edges. The selection of tile edges can affect the routing process and result in the generation of long wires. However, the internal edge only affects the routing within the tile node and has a minimal impact on the routing length. Third, compared with the cost of the internal edge, we are more concerned about the routing intersection caused by the selection of the internal edge. Therefore, we set *Q* to a minimum value to reduce model complexity and running time.

The multi-capacity fan-out WLCSP I/O ordered routing model evolved from the single-capacity model, with three key differences between them. First, a tile node can only allow one I/O to pass through in the single-capacity model, while in the multi-capacity model, the tile node is transformed into many internal boundary nodes to accommodate multiple I/Os. Second, in the single-capacity model, one fan-out bump is connected to one tile node by one edge, whereas in the multi-capacity model, multiple fan-out bumps are connected to one tile node through multiple edges; specifically, they are connected to the internal boundary nodes. Third, the internal edges of the tile nodes have separate weights *Q* in the multi-capacity model. However, to reduce the complexity of the model, we set *Q* to a minimum value, thereby ignoring the internal costs of the tile node.

The fan-out WLCSP I/O ordered routing problem can be transformed into a MMCF problem. It is described as follows: Under some constraints, find a feasible flow with the minimum cost to make *N* source points vs reach sink points (fan-out bump B1). Then, the feasible flow can be converted into the shortest routing path of *N* I/Os.

#### 3.1.3. Model Compactness

**Figure 6:**
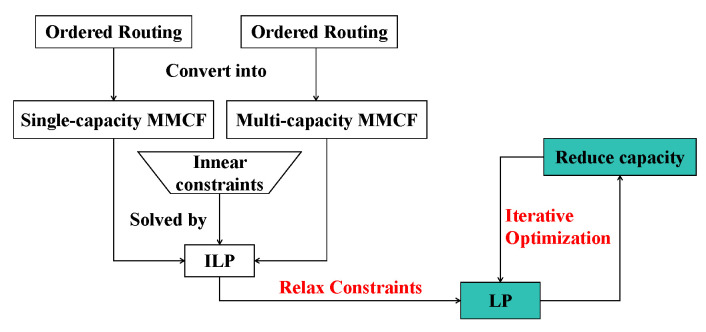
The change in our method compared with previous work.

To our knowledge, ref. [13] first proposed the single-capacity MMCF model, while ref. [14] introduced the multi-capacity MMCF model for ordered routing. Both studies utilized the ILP method to solve the MMCF model. However, when dealing with large-scale fan-out WLCSP ordered routing, the performance of ILP strategies suffers dramatically due to the increasing number of variables and time-consuming preprocessing. Therefore, we convert ILP into LP by relaxing the routing constraints to reduce the running time. Furthermore, we iteratively optimize the LP solution by reducing the edge and node capacities to decrease the number of wires in the congestion region. The difference is shown in Figure 6.

### 3.2. Preliminary Routing

This section proposes an LP solver for the fan-out WLCSP I/O ordered routing problem. We first introduce the traditional ILP solver for the ordered routing problem and then convert it into an LP solver to shorten the routing time. Finally, we use a DFS algorithm to obtain the candidate path set from the LP flow results.

#### 3.2.1. Fan-Out Ordered Routing ILP Solution

The traditional solution to the MMCF problem is ILP. The ILP formula includes the objective function to ensure all commodities flow smoothly from the source to the sink at the lowest cost. In addition to flow conservation and commodity value constraints that the MMCF model itself must consider, the ordered routing problem also needs to consider capacity, ordering, and non-crossing constraints. The ILP formula is as follows:

(1a) $$\begin{array}{cc} & \;\min\sum_{(q,r)\in E}\sum_{k\in DP}c^{e^{qr}}x_{k}^{e^{qr}},c^{e^{qr}}\in C \end{array}$$

(1b) $$\begin{array}{cc} s.t. & \;\sum_{q\in D}x_{k}^{e^{uq}}-\sum_{w\in D}x_{k}^{e^{wu}}=0,u\in DT \end{array}$$

(1c) $$\begin{array}{cc} & \;\sum_{w\in D}x_{k}^{e^{s_{k}w}}=1↔\sum_{w\in D}x_{k}^{e^{t_{k}w}}=1,s_{k}\in DP,t_{k}\in DI \end{array}$$

(1d) $$\begin{array}{cc} & \;\sum_{k\in K}x_{k}^{e^{qr}}\in \left\{0,1\right\},e^{qr}\in E,k\in K \end{array}$$

(1e) $$\begin{array}{cc} & \;\sum_{k\in K}x_{k}^{e^{qr}}\leq 1,e^{qr}\in E \end{array}$$

(1f) $$\begin{array}{cc} & \;\sum_{k\in K}\left(x_{k}^{e^{ar}}+x_{k}^{e^{br}}+x_{k}^{e^{cr}}\right)\leq 1,r\in DT \end{array}$$

(1g) $$\begin{array}{cc} & \;x_{j}^{e^{b_{n+1}b_{n}}}-x_{i}^{e^{b_{n+1}b_{n}}}\geq 0,b_{n}\in DI,1\leq i\leq j\leq N \end{array}$$

Formulation (1a) is the objective function whose goal is to guarantee that the network flow delivers the most I/Os for the least cost. Constraint (1b) and Constraint (1c) are the flow constraints to ensure that the solution of the LP equation is a complete flow path from the source point to the sink point. Constraint (1d) indicates that the decision variable $x_{k}^{e^{qr}}$ is a 0 – 1 variable. Constraint (1e), Constraint (1f), and Constraint (1g) are the tile edge capacity constraint, the tile node capacity constraint, and the ordered constraint. These three constraints are customized for ordered routing.

When facing the multi-capacity or large-scale ordered routing problems, the ILP solver takes too long to solve, limited by the model’s strict constraints and decision variable restrictions. Therefore, we will relax the decision variable $x_{k}^{e^{qr}}$ from an integer to a value between 0 and 1, simplify the ILP problem to an LP problem, and reduce the difficulty of solving large-scale, multi-capacity ordered routing problems.

#### 3.2.2. Fan-Out Ordered Routing LP Solution

(1) Setting capacity: We introduce the tile edge capacity $a^{r}$ and node capacity $a^{e^{qr}}$. When congested candidate routing results appear, we can iterate the LP solution by adjusting the capacity of the tile edge and node of the congested area to obtain a better candidate routing result set. When a part of the I/O is successful in the routing, the remaining capacity of the edges and nodes of the routing path is adjusted to 0 to realize the active obstacle avoidance function. By scaling the capacity and iterating the routing, our method can effectively address congestion issues and optimize routing quality.

(2) LP solution: We relax the value of the decision variable $x_{k}^{e^{qr}}$ to any value from 0 to 1 and reduce the number of LP constraints by placing non-crossing constraints into Path Choosing for subsequent treatment, which significantly reduces the difficulty and time of solving the problem. In addition, the capacity limits $a^{r}$ and $a^{e^{qr}}$ are added for subsequent iterative routing. The edge and node capacity start from 1. It is adjusted according to the routing results in subsequent iterative routing. The LP formula finally based on the directed graph G(D,E,C,V) is as follows:

(2a) $$\begin{array}{cc} & \;\min\sum_{(q,r)\in E}\sum_{k\in DP}c^{e^{qr}}x_{k}^{e^{qr}},c^{e^{qr}}\in C \end{array}$$

(2b) $$\begin{array}{cc} s.t. & \;\sum_{q\in D}x_{k}^{e^{uq}}-\sum_{w\in D}x_{k}^{e^{wu}}=0,u\in DT \end{array}$$

(2c) $$\begin{array}{cc} & \;\sum_{r\in D}x_{k}^{e^{s_{k}r}}=1↔\sum_{w\in D}x_{k}^{e^{t_{k}w}}=1,s_{k}\in DP,t_{k}\in DI \end{array}$$

(2d) $$\begin{array}{cc} & \;\sum_{k\in K}x_{k}^{e^{qr}}\in \left[0,1\right],e^{qr}\in E,k\in K \end{array}$$

(2e) $$\begin{array}{cc} & \;\sum_{k\in K}x_{k}^{e^{qr}}\leq a^{e^{qr}},e^{qr}\in E,a^{e^{qr}}\in VE \end{array}$$

(2f) $$\begin{array}{cc} & \;\sum_{k\in K}\left(x_{k}^{e^{ar}}+x_{k}^{e^{br}}+x_{k}^{e^{cr}}\right)\leq a^{r},r\in DT,a^{r}\in VN \end{array}$$

(2g) $$\begin{array}{cc} & \;Mx_{j}^{e^{b_{n+1}b_{n}}}-x_{i}^{e^{b_{n+1}b_{n}}}\geq 0,b_{n}\in DI,1\leq i\leq j\leq N \end{array}$$

Formula (2a) is the objective function, which aims to ensure that the routing of the most pre-assignment I/Os is completed at the lowest cost in the fan-out WLCSP I/O ordered routing problem. *C* is the set of all directed edge costs divided into CO and CI in the multi-capacity ordered routing model. CO refers to the cost of directed edges between tile nodes. CI refers to the cost of internal edges. We set $c^{e^{qr}}=1$ and Q≪1.

Constraint (2b) is the flow conservation. When I/O *k* passes through any tile node *u*, the inflow should be the same as the value of the outflow, ensuring that the pre-assignment I/O flow values are balanced, leaving and entering each tile node. Constraint (2b) is the most essential constraint of the network flow model.

Constraint (2c) sets the value of 1 for each pre-assignment I/O and targets the arrival from the source $s_{i}$ to the sink $t_{i}$. Since constraint (2b) ensures that the in-degree and out-degree of all tile nodes except source $s_{i}$ and sink $t_{i}$ are balanced, it is guaranteed that each path reaches sink $t_{i}$ continuously.

Constraint (2d) is an LP constraint that requires that the flow value of the pre-assignment I/O in the tile edge and tile node can be any value from 0 to 1, which means that as long as $x_{k}^{e^{qr}}$>0, the I/O *k* is considered to pass through the edge $e^{qr}$. Therefore, the LP method’s pre-assignment I/O routing result is not unique. The flow values for pre-assignment I/O in tile edges and nodes can only be 1 or 0 in ILP. When $x_{k}^{e^{qr}}=0$, it indicates that IO *k* does not need to be routing on edge $e^{qr}$. When $x_{k}^{e^{qr}}=1$, it indicates that edge $e^{qr}$ is exclusively occupied by IO *k*, and other I/O cannot routing on edge $e^{qr}$.

Constraint (2e) is the tile edge capacity constraint. We set the total capacity of the pre-assignment I/O flow values passing through the tile edge, which does not exceed $a^{e^{qr}}$.

In Constraint (2f), before the MMCF model is optimized, each intermediate node is connected to the four surrounding nodes to generate four directed edges in different directions. After optimization, the intermediate node is only connected to the three surrounding nodes, generating three directed edges in different directions. We will set a,b,c nodes from the tile node *r* in three directions. The constraint ensures that the sum of the pre-assignment I/O flow values flowing into tile node *r* is not greater than the node capacity limit $a^{r}$.

The ordered constraint is (2g), where N is the number of pre-assignment I/Os, *i* and *j* are two I/Os, and the I/O *j* pre-assignment order is greater than *i*. In Figure 5b, we number the boundary bumps and connect them counterclockwise to B1 as the end pad of the ordered routing flow model. Constraint (2g) requires that when the path of I/O *i* flows through the boundary edge $e^{b_{n+1}b_{n}}$, I/O *j* must also flow through the boundary edge $e^{b_{n+1}b_{n}}$, and it finally reaches the end point B1. Constraint (2g) ensures the ordered requirement is satisfied between each I/O path. Constraint (2g) adds a constant *M* in LP. The value of *M* needs to be large enough that no matter how small $x_{j}^{e^{b_{n+1}b_{n}}}$ is, as long as it is greater than 0, constraint (2g) can be successfully constrained. The value range of M is typically from 2 to 10 times the number of I/Os in practical applications. The larger the scale of the routing problem, the greater the multiplier will be.

#### 3.2.3. Ordered Routing Candidate Result Generation

**Figure 7:**
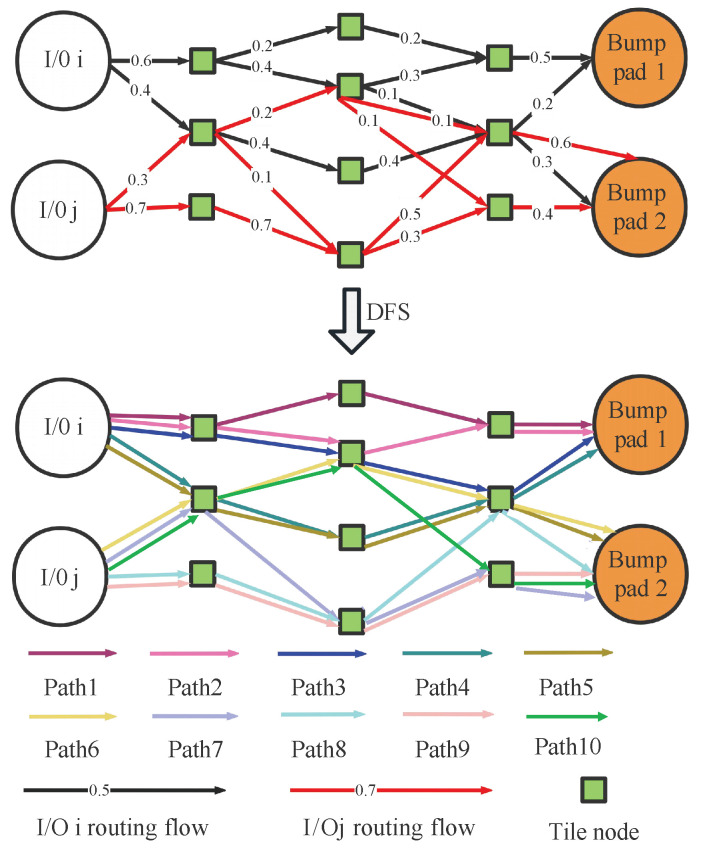
Diagram of the LP flow result conversion routing path.

After solving the LP equation, we can obtain the flow value distribution for each pre-assignment I/O routing, including all pre-assignment I/Os across the entire flow model. However, as shown in Figure 7, it is presented as an LP flow result and cannot be directly used as a routing result. Therefore, we use DFS to convert the LP flow result of pre-assignment I/Os routing into the candidate routing paths. The algorithm is shown in Algorithm 1.

Figure 7 is a diagram of the LP flow result conversion routing path. The first path that I/O *i* finds is Path2, and it subtracts the minimum flow 0.3 from the tile nodes and edges capacity along the routing path. Then, Algorithm 1 will search for Path5, Path1, Path3, Path4 in sequence. The path of I/O *j* is (Path8-Path6-Path10-Path9-Path7). The pre-assignment I/O paths obtained by LP and the DFS algorithm have different I/O path crossings, such as Path5 and Path6. Therefore, the final routing result needs to be further refined to address the path crossing problem between different I/Os.

### 3.3. Path Choosing

The candidate routing result set obtained from the flow result of the LP formula does not consider the non-crossing constraint. Path choosing involves selecting the legal routing result with the shortest total routing length and non-crossing paths from the candidate routing result set. This section discusses the non-crossing constraints in the MMCF model, path-crossing graph, and heuristic algorithm to obtain legal routing results from the candidate routing result set.
**Algorithm 1** DFS**Require:** The directed graph: G(D,E,C,V), The pre-assignment I/Os set: *K*, The set of $x_{k}^{e^{qr}}$: *X***Ensure:** 
*n* path sets: P1, P2, …, Pn  1:**while **K≠⌀** do**  2:    Take a I/O *i* out of set *K*.  3:    **while** true **do**  4:        Push the node where I/O *i* is located to stack *Q*  5:        **while** Q≠⌀ **do**  6:           currentNode = pop stack from top  7:           findpath = false  8:           **if** currentNode is the fan-out bump **then**  9:               Reverse the search process to obtain path *p* and push *p* into Pi10:               Find the smallest flow $f_{min}$ on the path *p*11:               Subtract $f_{min}$ from the flow of all edges on path *p*12:               findpath = true13:               Break14:           **end if**15:           Visit all the neighbor edges of currentNode in descending order of flow(exclude the zero-flow edge)16:           Obtain neighbor node through neighboring edges17:           **if** the neighbor node is not marked **then**18:               mark neighbor node and push it into stack *Q*19:           **end if**20:        **end while**21:        **if** findpath == false **then**22:           Break23:        **end if**24:    **end while**25:    Remove I/O *i* from set *K*26:**end while**27:**return** P1, P2, …, Pn.

#### 3.3.1. Crossings in MMCF Model Introduction

**Figure 8:**
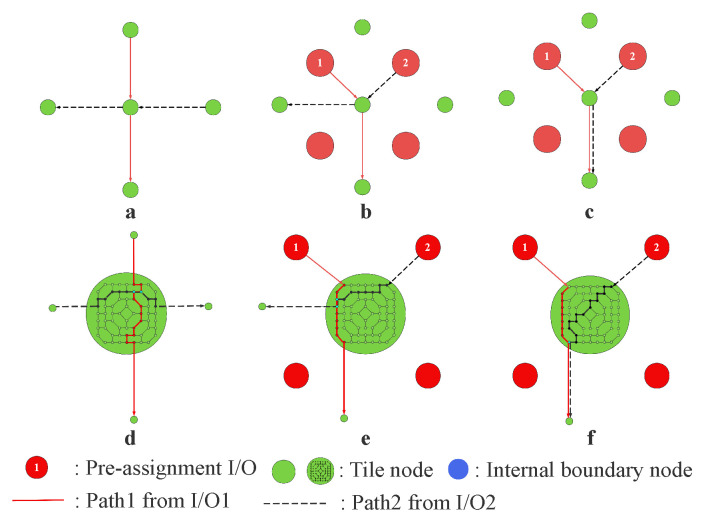
(**a**) the node crossing where two routing paths cross at a tile node in the single-capacity model. (**b**) the node crossing of two routing paths cross at the I/O in the single-capacity model. (**c**) the edge crossing of the single-capacity model. (**d**) the node crossing where two routing paths cross within the tile node in the multi-capacity model. (**e**) the node crossing of two routing paths cross at the I/O in a multi-capacity model. (**f**) the edge crossing of the multi-capacity model.

In the candidate routing result set, when different pre-assignment I/O paths route the same tile node or edge, it causes path crossing. We divide crossings into two types: node crossing and edge crossing, as shown in Figure 8. We define the node crossing as two routing paths that cross each other at the tile node or within the tile node. Node crossing will lead to a short circuit (DRC-short). We define the edge crossing as two routing paths passing through the same tile edge simultaneously. Edge crossing will violate the wiring spacing rules, preventing manufacturers from completing production.

We assign different weights to the two types of crossing: edge crossing weight $W_{b}=10$ and node crossing weight $W_{i}=1$. The weights $W_{b}$ and $W_{i}$ can be adjusted according to the ratio of the two crossings. If the percentage of the node crossing to the total crossing is greater than 35%, we will increase $W_{i}$. If the proportion of edge crossings is greater than 80%, increase $W_{b}$. However, the weight $W_{b}$ must be greater than $W_{i}$ because the effect of edge crossing is more significant than node crossing. On the one hand, edge crossing will directly lead to a loss of production. On the other hand, once the edge crossings are resolved, many node crossings will disappear. Therefore, assigning a greater weight to edge crossing not only aligns with the actual situation but also accelerates convergence.

#### 3.3.2. MMCF Non-Crossing Constraint Solution

**Figure 9:**
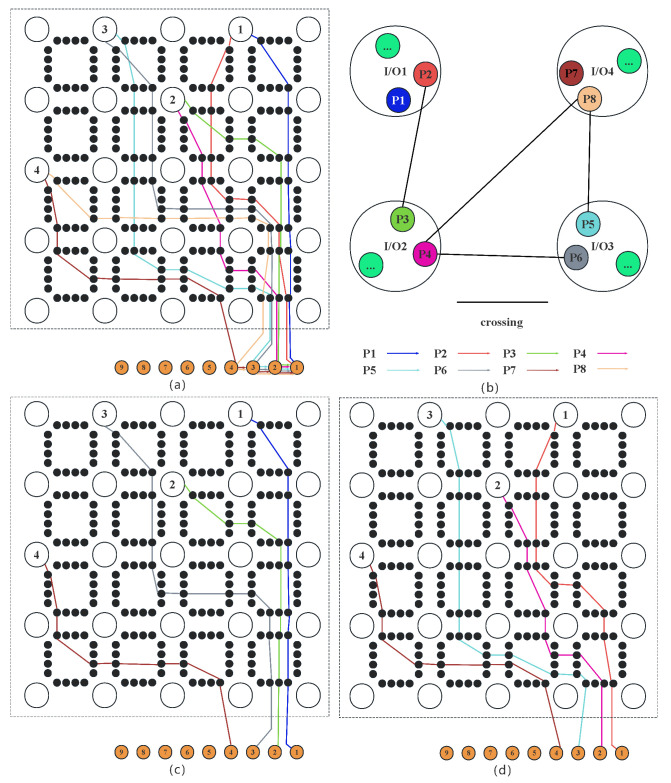
Diagram of the non-crossing constraint solution process of the MMCF model. (**a**) shows that each I/O has two candidate paths. (**b**) is the constructed crossing graph. (**c**) is the optimal solution obtained by ILP. (**d**) is the solution obtained by the weighted heuristic algorithm.

In the multi-capacity MMCF model, as shown in Figure 9a, each of the I/Os has two different candidate paths. There are many node crossings and edge crossings between the paths, such as P4, P5, P6, and P8. If we choose the best path for all pre-assignment I/Os, the crossing weight should be reduced to a tiny value or zero. Therefore, the problem to be solved in Path Choosing is to select a path from each pre-assignment I/O routing path set to form the final routing result with the minimum crossing weight. We proposed a weighted heuristic algorithm to solve this problem, as shown in Algorithm 2.

First, we randomly select a path from each pre-assignment I/O candidate path set as the initial routing result, denoted as $T_{1}$. Then, we calculate the crossing weights between the *N* paths in $T_{1}$ and add them up to obtain the total crossing weight $W_{T_{1}}$. The remaining F−N paths are denoted as $F^{\prime }$. Afterwards, we randomly select path *r* from the set $F^{\prime }$, replace *r* with the path of the same I/O in $T_{1}$, obtain a new path set $T_{2}$, and start calculating the total crossing weight $W_{T_{2}}$ of the *N* paths in $T_{2}$. If $W_{T_{2}}$ is less than $W_{T_{1}}$, assign $W_{T_{2}}$ to $W_{T_{1}}$, and replace $T_{1}$ with $T_{2}$. After the replacement is successful, we remove path *r* from the set $F^{\prime }$, return to step 4, and start the loop until the set $F^{\prime }$ is empty. After jumping out of the loop, Algorithm 2 outputs the path set $T_{1}$ with the smallest $W_{T_{1}}$.
**Algorithm 2** Optimize Path Set**Require:** 
The set of candidate paths *F* for all pre-assignment I/Os, number of I/Os *N***Ensure:** 
The optimal path set $T_{1}$ with minimal total crossing weight $W_{T_{1}}$  1:Initialize $T_{1}$ with *N* paths randomly selected from *F*  2:$W_{T_{1}}\leftarrow$ calculate total crossing weight of $T_{1}$  3:$F^{\prime }\leftarrow F-T_{1}$  4:**while **$F^{\prime }$ is not empty **do**  5:    Select path *r* from $F^{\prime }$  6:    Replace old_path of the same I/O as *r* in $T_{2}$ with *r*  7:    $W_{T_{2}}\leftarrow$ calculate total crossing weight of $T_{2}$  8:    **if** $W_{T_{2}}<W_{T_{1}}$ **then**  9:        $W_{T_{1}}\leftarrow W_{T_{2}}$10:        $T_{1}\leftarrow T_{2}$11:    **end if**12:    $F^{\prime }\leftarrow F^{\prime }-r$13:**end while**14:**return **$T_{1},W_{T_{1}}$

Using ILP to solve the MMCF problem can ensure obtaining the global optimal solution, as shown in Figure 9c. The optimal path selection is (P1, P3, P6, P7) with a wiring length of 18. The results obtained by using Algorithm 2 to solve the crossing problem caused by LP are shown in Figure 9d. The path selection is (P2, P4, P5, P7) with a wiring length of 19. Algorithm 2 is weighted sensitive and random. Therefore, it is prone to getting stuck in local optimal solutions. Compared with the ILP method, our algorithm achieves a faster routing speed and a larger solution scale, while the loss is a very small increase in line length.

After completing the non-crossing constraint path screening, several cases exist in the path set $T_{1}$. One is $W_{T_{1}}$ = 0, which means that there is no intersection between the routing results, achieving 100% accessibility, which means that an optimal solution has been found; the other is that $W_{T_{1}}$ > 0, which means there is still an crossing between the path set $T_{1}$.

#### 3.3.3. Iterative Routing Settings

Some pre-assignment I/O paths in $T_{1}$ do not cross, which is the non-crossing routing result for setting it as $T_{1}^{n}$. In the iterative routing process, we remove the I/O that has been routed from the pre-assignment I/O set and set the remaining capacity of the tile node and edge used by the path set $T_{1}^{n}$ that has been routed to 0, which ensures other pre-assignment I/Os can actively avoid obstacles during the iterative routing process and do not cross with $T_{1}^{n}$. Some pre-assignment I/O paths in $T_{1}$ have a crossing area. During the iterative routing process, we will continuously reduce the tile node and edge capacities in the crossing area phase by phase to optimize the routing results in the crossing area.

There are two exit conditions in the iterative routing process. One is $W_{T_{1}}$ = 0, which means that there is no crossing between the routing results, and 100% reachability is achieved. The other is that the tile node and edge reach the capacity threshold during the iterative routing process. This means that the path can no longer be optimized or that the time cost of optimization is too high. Therefore, it is unnecessary to continue optimization and exit the cycle. We set the capacity threshold at 0.375 and have an adjustable range of 0.35–0.4. When there is less congestion, we will increase the threshold to reduce the number of iterations and achieve less running time. When the routability is low, we will appropriately decrease the threshold to achieve more iterations and find a better solution.

#### 3.3.4. Discussion on Computational Cost

Reference [15] has already demonstrated that ILP and ordered routing problems are NP-complete (Non-deterministic Polynomial-time Complete). Reference [14] demonstrates that the solution complexity of the multi-capacity MMCF model is a linear multiple of the ILP problem. As the number of nodes and edges in the commodity flow graph increase rapidly, the computational cost of solving the problem using ILP has grown exponentially. Our method breaks down the fan-out WLCSP I/O ordered routing problem into three sub-stages and respectively adopts LP, DFS, and a weight-based heuristic algorithm to solve them. We constructed the LP formula of the MMCF model and used Gurobi optimization [16] to solve it with the time complexity of O(mn). *m* represents the number of constraints and *n* represents the total number of variables. The time complexity of DFS is O(V+E), where *V* represents the number of tile nodes and *E* represents the number of tile edges. The time complexity of the heuristic algorithm is O(P), where *P* represents the total number of candidate paths. Therefore, the total time complexity of our method is polynomial time. When dealing with large-scale ordered routing problems, the exponential running time for solving ILP is unacceptable. Our method can be solved in polynomial time, addressing the computational time problem of existing algorithms.

## 4. Experimental Results

In this paper, we propose an ordered routing algorithm with low time complexity and strong feasibility that applies the MMCF model to the ordered routing problem of fan-out WLCSP. We implement our algorithm in C++ and test it on a workstation with a 2.30 GHz Intel Core i7 processor and 16 GB of memory. We reproduce the constraint-driven ConDri(P) model with a partitioning strategy and a multi-capacity ordered routing model with a partition strategy (WRDPS) in ILP [9,13,14]. We test some cases, including cases (Case 1–Case 3, Case 5, Case 6, Case 8–Case 12) collected from various academic works [14,17]. In addition, we construct two special cases. We transformed a large-scale industrial case into Case 4. This case has a large number of grids and an uneven I/O distribution. The IO distribution in some locations is very dense. Professional engineers must use long and winding wires to complete routing. Moreover, we randomly generated 20 I/Os to prevent the routing area of this case from being decomposed into multiple sub-areas. We modify Case8 to obtain Case 7, demonstrating the performance of our algorithm. We reduce the size of Case 8 by half and relocate some of the IOs that are originally located at the boundary to the middle of the routing area, thereby increasing the difficulty of routing. Solvers in the algorithm use the Gurobi optimization solver [16].

We conduct experiments on all the cases more than ten times. We take the best experiment as the final result and calculate the average running time of all experiments as the final routing time to eliminate the instability of CPU load. All experiments are simulation-based, and validation in real design flow is planned for future work.

### 4.1. Results and Comparisons

Table 2 concerns the single-capacity ordered routing problem. The different groups adopt the traditional ILP method, and the ILP method is accelerated by ConDri(P), a compact model of the ordered path problem driven by linear constraints that eliminate infeasible solutions, significantly reducing the redundant constraints in ILP. Table 2 includes the case size (rows, cols), number of pre-assignment I/Os, distribution type of I/Os, routing length (Length), time cost (Time), and routing success rate (Ratio). As seen from Table 2, our method can complete the routing scale twice as large as the traditional ILP method in the single-capacity routing problem.

Table 3 concerns the multi-capacity ordered routing problem. The different groups adopt the traditional ILP method and the accelerated ILP method using the wiring resource driven partition strategy (WRDPS). WRDPS is based on the candidate terminal nodes, and the ordered routing problem is divided into several small-scale ordered routing sub-problems. The difference between Table 2 and Table 3 is that $C_{b}$ represents the capacity size. As can be seen from Table 3, our algorithm can solve all cases, and our average routing time is reduced by 17% in the multi-capacity ordered routing problem, except for case 7.

**Figure 10:**
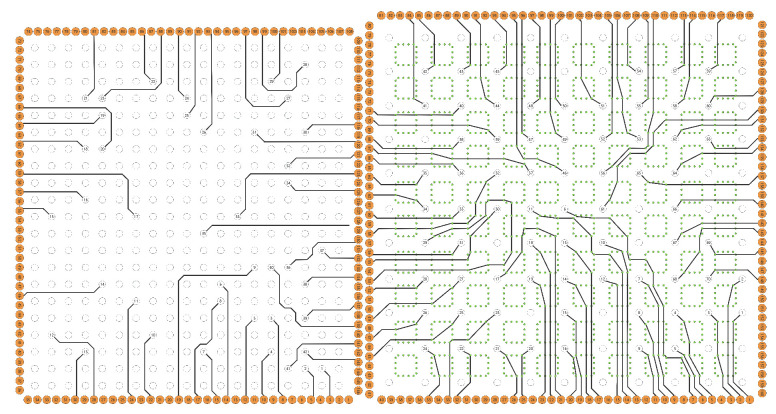
Single-capacity ordered routing result of Case 2 (**left**) and multi-capacity ordered routing result of Case 7 (**right**).

Figure 10 (right) shows the routing result of our method for Case 7. Case 7 has a high I/O distribution density and routing model capacity. The small number of boundary I/Os results in a poor WRDPS acceleration effect, and the scale of sub-problems is too large, which renders the traditional ILP unable to solve the problem. Our method is not affected by the number of boundary I/O distributions. It is the polynomial time complexity and handles problems with higher I/O distribution density. Finally, our method obtains legal routing results within 1 h. Figure 10 (left) shows the routing result of our method for Case2. Under the premise that the routing result meets various constraints, our method adjusts the tile node and edge capacity. It iterates the routing multiple times to obtain a routing result with a more uniform distribution of routing paths.

### 4.2. Analysis of Routing Time

**Figure 11:**
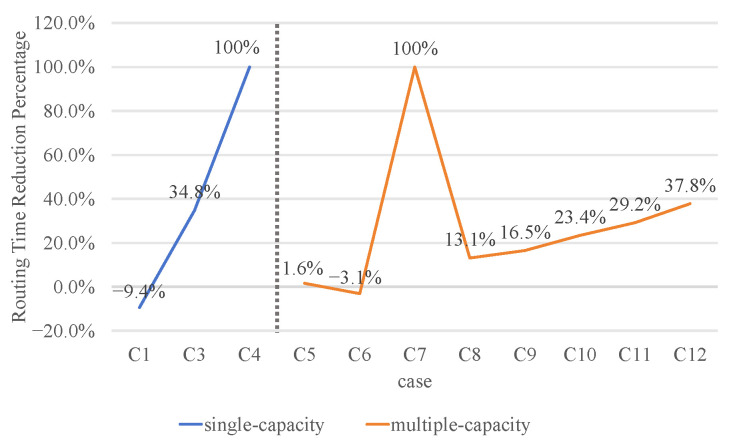
The routing time reduction percentage compared with ILP(ConDri(P)) and MC-MCF (WRDPS) in single-capacity and multiple-capacity ordered routing.

Figure 11 compares the time spent by ILP(ConDri(P)), MC-MCF (WRDPS), and our method in the ordered routing problem. The blue line in Figure 11 represents the time reduction percentage compared between our method and ILP(ConDri(P)) in solving the single-capacity ordered routing problem. The red line in Figure 11 represents the time reduction percentage compared between our method and MC-MCF (WRDPS) in solving the multi-capacity ordered routing problem. 100% represents that the time spent is greater than one time, and a negative value indicates that our method takes more time.

Compared to the ILP method, our method will take longer to route in single-capacity small-scale ordered routing problems. In Case 2, the routing time of ILP(ConDri(P)) is much shorter than our method. Because Case 2 is perfectly divided into four sub-problems by the acceleration strategy, the difficulty of solving the ILP and the routing time is significantly reduced. Our method requires multiple iterations in the ordered routing process, which increases the time spent. As the scale increases, the time spent on our method is shorter than that of ILP methods. Because our method has low complexity, it does not become unsolvable due to the explosive growth of computational costs as the scale and capacity of ordered routing increase. Our method can produce a high-performance solution to single-capacity large-scale ordered routing problems, such as Case 4.

Our method has generally become more advantageous in handling multi-capacity ordered routing problems as the scale and capacity increase. In Case 6, the MC-MCF (WRDPS) method has a better acceleration effect. However, in Case 7, MC-MCF (WRDPS) cannot divide the case into sub-problems that are easy to handle with ILP, resulting in its inability to solve it. Our method successfully obtains legal routing results by relaxing decision variables and reducing constraints. Compared with the WRDPS, our method can reduce the routing time by 17% in multi-capacity ordered routing.

### 4.3. Analysis of Routing Length

**Figure 12:**
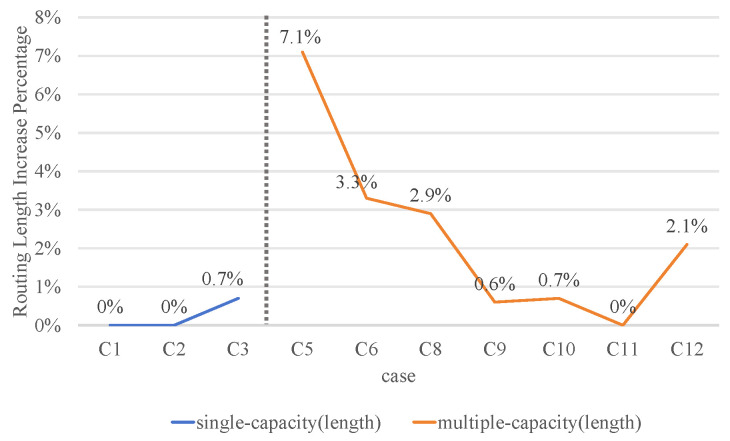
The routing length increase percentage compared with ILP(ConDri(P)) and MC-MCF (WRDPS) in single-capacity and multiple-capacity ordered routing.

Figure 12 compares the routing length in ILP(ConDri(P)), MC-MCF (WRDPS), and our method. As shown in Figure 12, our method exhibits a longer routing length for both single-capacity and multi-capacity ordered routing. The routing length will increase by 3% compared with the MC-MCF (WRDPS) method in multi-capacity ordered routing. Our method uses a heuristic algorithm to obtain the final routing result. Because the heuristic algorithm is a selective strategy, it cannot guarantee the optimal solution. The advantage of the heuristic algorithm is that it can improve the running speed with minimal cost. In the actual fan-out WLCSP design process, 3% line length fluctuation is acceptable. Our algorithm can effectively improve the scale of the ordered routing problem, which aligns more closely with the current trend of wafer-level chip development. It can also reduce the time required for solving the RDL multi-capacity ordered routing.

### 4.4. Runtime Breakdown Analysis

**Figure 13:**
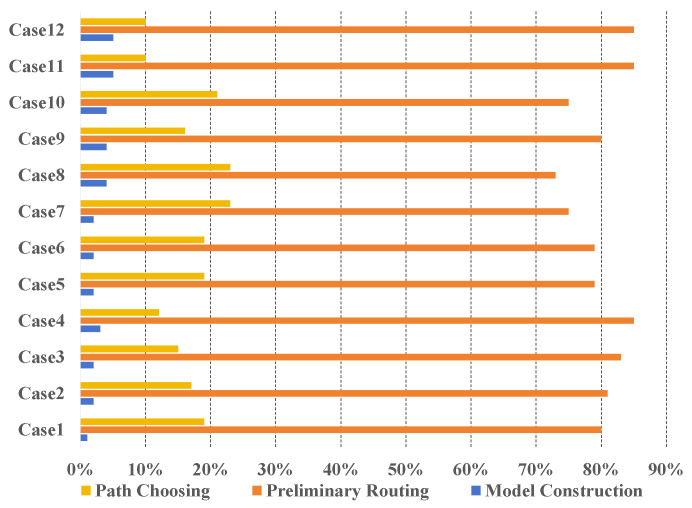
The running time ratio of each part in our algorithm.

Figure 13 shows the runtime breakdown of our algorithm. Because the fan-out WLCSP I/O ordered routing model is built only once, it accounts for 3%. As the scale of the routing problem increases, the proportion of model construction is also rising. Preliminary routing is the core of our algorithm. We solve the LP equation and transform the flow result into the routing path in the Preliminary routing. In addition, Preliminary routing requires multiple iterations and optimizations. Therefore, Preliminary routing accounts for 80%. Path choosing adopts the heuristic algorithm to handle the problem of path intersection. When the IO distribution is dense and routing is complex, the proportion of runtime on path choosing will increase. On average, path choosing accounts for 17%.

## 5. Conclusions

We proposed a method of LP and crossing weight heuristic algorithm to solve the fan-out WLCSP I/O ordered routing problem. First, the MMCF model was constructed based on the distribution of I/O and bump pads. Next, the LP solver replaced the traditional ILP solver to handle larger-scale and larger-capacity RDL ordered routing problems. Then, the DFS method converted the LP method’s pre-assignment I/O path flow results into a candidate path set. We set the crossing weights for different I/O paths when they crossed and propose a crossing weight heuristic algorithm to obtain legal routing results. In the iterative routing, the tile node and edge capacity of the already routed I/O path were reset to 0 to avoid crossing completed I/O paths. Experimental results show that our algorithm can solve twice the scale of the RDL ordered routing problems and reduce the routing time by 17% when dealing with multi-capacity RDL ordered routing problems. Meanwhile, the accuracy loss of LP and heuristic algorithms will lead to a 3% increase in the wiring length. It is valuable in the actual chip production to exchange the wiring length metric with minimal loss for faster routing speed and larger solution scale.

## 6. Outlook

In the future, we plan to improve the proposed method, including the following three points. First, we plan to combine the method with business tools to verify its feasibility in the actual fan-out WLCSP I/O ordered routing problem. Secondly, we plan to apply graph neural networks to learn the features of different cases and enhance the capabilities of heuristic algorithms through different features. Thirdly, we plan to apply reinforcement learning to guide the rip-up and reroute and improve the final routing result.

## Figures and Tables

**Table 1:** Nomenclature and abbreviations.

Symbols and Descriptions		Abbreviations and Full Names	
G(D,E,C,V)	Network flow model	WLCSP	Wafer-level chip-scale packaging
Bi	Fan-out bump with order *i*	ILP	integer linear programming
*N*	Number of pre-assignment I/Os	RDL	redistribution layer
*u*	Tile node	MMCF	min-cost multi-commodity flow
$u_{b}$	Internal boundary node	LP	linear programming
$u_{in}$	Internal filling node	DFS	depth-first search
$e_{in}^{u}$	Internal edge	IC	integrated circuit
$e^{qr}$	Edge from node *q* to *r*	FA routing	free-assignment routing
$x_{k}^{e^{qr}}$	Flow of I/O *k* in edge $e^{qr}$	PA routing	pre-assignment routing
$c^{e^{qr}}$	Cost of tile edge $e^{qr}$	UA routing	unified-assignment routing
*Q*	Cost of internal edge		
$a^{e^{qr}}$	Capacity of edge $e^{qr}$		
$a^{r}$	Capacity of node *r*		
$s_{i}$, $t_{i}$	Source and sink nodes		
$e^{b_{n+1}b_{n}}$	Boundary edge from $B_{n+1}$ to $B_{n}$		
$T_{n}$	Set of all paths		
$W_{T_{n}}$	Total crossing weights of set $T_{n}$		
$W_{b}$	edge crossing weight		
$W_{i}$	node crossing weight		

**Table 2:** Experimental results for single-capacity ordered routing problem.

Cases	Cols	Rows	I/Os	Type	ILP			ILP(ConDri(P))			Our Method		
					Length	Time (s)	Ratio (%)	Length	Time (s)	Ratio (%)	Length	Time (s)	Ratio (%)
Case 1	8	6	10	3-side	19	0.13	100%	20	0.53	100%	20	0.58	100%
Case 2	20	21	42	4-side	76	64.87	100%	78	4.87	100%	78	31.96	100%
Case 3	30	30	100	4-side	/	/	/	720	32.65	100%	725	21.29	100%
Case 4	100	100	320	4-side	/	/	/	/	/	/	6168	146.92	100%

**Table 3:** Experimental results for multi-capacity ordered routing problem.

Cases	Cols	Rows	I/Os	Capacity	MC-MCF			MC-MCF (WRDPS)			Our Method		
					Length	Time (s)	Ratio (%)	Length	Time (s)	Ratio (%)	Length	Time (s)	Ratio (%)
Case 5	8	8	43	2	98	45,895	100%	98	183	100%	105	180	100%
Case 6	10	10	32	2	/	/	/	90	158	100%	93	163	100%
Case 7	11	11	70	4	/	/	/	/	/	/	255	220	100%
Case 8	20	20	171	4	/	/	/	470	746	100%	484	648	100%
Case 9	23	24	93	2	/	/	/	338	206	100%	340	172	100%
Case 10	24	24	93	2	/	/	/	413	1171	100%	416	896	100%
Case 11	30	30	158	3	/	/	/	641	360	100%	641	255	100%
Case 12	50	50	300	2	/	/	/	1617	2032	100%	1652	1264	100%

## Data Availability

The original contributions presented in this study are included in the article. Further inquiries can be directed to the corresponding author.
